# A Selective RAG-Enhanced Hybrid ML-LLM Framework for Efficient and Explainable Fatigue Prediction Using Wearable Sensor Data

**DOI:** 10.3390/bioengineering13010058

**Published:** 2026-01-03

**Authors:** Soonho Ha, Taeyoung Lee, Hyungjun Seo, Sujung Yoon, Hwamin Lee

**Affiliations:** 1 Department of Medical Informatics, College of Medicine, Korea University, Seoul 02841, Republic of Korea; 2 Ewha Brain Institute, Ewha Womans University, Seoul 03760, Republic of Korea

**Keywords:** fatigue prediction, wearable sensors, machine learning, large language model (LLM), hybrid inference, explainable AI

## Abstract

Fatigue is a multifactorial phenomenon affecting both physical and psychological performance, particularly in high-stress occupations. Although wearable sensors enable continuous monitoring, conventional machine-learning (ML) models can produce unstable, weakly calibrated, and opaque predictions in real-world settings. To improve reliability and interpretability, we developed a selective Retrieval-Augmented Generation (RAG)–enhanced hybrid ML–LLM framework that integrates the efficiency of ML with the reasoning capability of large language models (LLMs). Using wearable and ecological momentary assessment data from 297 emergency responders (9543 seven-day windows), logistic regression, XGBoost, and LSTM models were trained to classify fatigue levels dichotomized by the median of daily tiredness scores. The LLM was selectively activated only for borderline ML outputs (0.45 ≤ *p* ≤ 0.55), using symbolic rules and retrieved analog examples. In the uncertainty region, performance improved from 0.556/0.684/0.635/0.659 to 0.617/0.703/0.748/0.725 (accuracy/precision/recall/F1). On the full test set, performance similarly improved from 0.707/0.739/0.918/0.819 to 0.718/0.741/0.937/0.827, with gains confirmed by McNemar’s paired comparison test (*p* < 0.05). SHAP-based ML interpretation and LLM reasoning analyses independently identified short-term sleep duration and heart-rate variability as dominant predictors, providing transparent explanations for model behavior. This framework enhances classification robustness, interpretability, and efficiency, offering a scalable solution for real-world fatigue monitoring.

## 1. Introduction

Fatigue represents a pervasive yet often underrecognized determinant of both mental health and occupational performance. Persistent fatigue has been linked to impaired cognitive functioning, reduced alertness, and increased risk of errors and accidents—particularly in high-demand occupations such as emergency response and healthcare [1,2]. In mental health contexts, chronic fatigue not only co-occurs with depressive and anxiety disorders but also predicts relapse and diminished treatment response [3,4]. Despite its clinical and societal significance, fatigue is inherently dynamic, fluctuating within and across days in response to sleep, workload, and environmental stressors. Capturing within- and between-day fatigue trajectories in naturalistic settings is essential for early detection, targeted intervention, and prevention of downstream mental-health deterioration [5,6].

Emergency responders (e.g., firefighters and police investigators) work under conditions of frequent acute stress exposure and irregular or rotating shift schedules [7,8]. These occupational demands lead to circadian rhythm disruption and cumulative fatigue, which, in turn, increase the risk of chronic deterioration in mental health [9,10]. Traditional single-timepoint or self-report fatigue assessments are inadequate to capture such rapid, within-day fluctuations in physiological and psychological states. To address these limitations, Ecological Momentary Assessment (EMA) has emerged as a promising approach that repeatedly evaluates fatigue and related affective indicators in real-world contexts, thereby reducing recall bias and enabling high-resolution temporal tracking [11,12].

Yet, despite its methodological strengths, EMA still faces practical challenges such as high participant burden, irregular response patterns, and frequent missingness, which hinder long-term adherence and limit its utility for predictive modeling [13,14,15,16]. These limitations have motivated the exploration of passive sensing approaches using wearable devices, which can continuously and unobtrusively capture physiological and behavioral markers related to fatigue—such as heart rate, heart rate variability (HRV), activity levels, and sleep patterns [17,18,19].

In this context, machine learning (ML) models have been increasingly applied to predict fatigue from wearable-derived physiological and behavioral features [20,21]. These models can efficiently learn multivariate relationships and provide quantitative predictions; however, they often behave as black boxes and may yield unreliable outputs under uncertain conditions, such as missing data, sensor drift, or atypical physiological patterns [22]. On the other hand, large language models (LLMs) excel in contextual reasoning and can integrate multiple cues, but their computational cost and latency limit real-time scalability when applied to every case [23,24,25]. To address this gap, we introduce a Selective Retrieval-Augmented Generation (RAG)-Enhanced Hybrid ML–LLM Framework that strategically combines the strengths of both paradigms. In this framework, an ML model provides fast and reliable predictions for most cases, while an LLM is selectively activated only when the ML prediction exhibits low confidence.

## 2. Materials and Methods

### 2.1. Study Population

This study utilizes wearable sensor and EMA data collected from November 2024 to March 2025. Wearable data were collected using the Xiaomi Smart Band 9. A total of 304 emergency responders (firefighters and police investigators) are enrolled as part of an ongoing project investigating fatigue and mental health among high-stress occupational groups, and all participants are recruited through the Ewha Brain Institute, Ewha Womans University. This study is conducted in accordance with the Declaration of Helsinki and is approved by the Institutional Review Board (IRB) of Ewha Womans University (IRB No. 2024-0258, approval date: 14 October 2024).

After excluding individuals with missing wearable sensor data (n = 6) or incomplete survey responses (n = 2), and resolving one overlapping case, the final analytic cohort consists of 297 participants (Supplementary Materials Figure S1). Participants are allocated to the training (n = 237) and test (n = 60) sets using a participant-level stratified split, ensuring comparable label distributions across splits. This results in 7781 and 1863 seven-day, day-level observation windows in the training and test sets, respectively (approximately 32.8 and 31.1 samples per participant).

To prevent any information leakage, data splitting is performed at the participant level prior to generating seven-day windows. All windows from the same individual are assigned exclusively to a single split (train, validation, or test), ensuring that no temporal or participant-overlapping information propagates across partitions.

Daily fatigue levels are assessed using the visual analogue scale for tiredness (VAS-tiredness), which consists of the item “How tired did you feel today?” [26]. Participants indicate their level of fatigue on a linear visual analog scale ranging from 0 to 100, where higher scores indicate greater subjective fatigue.

For modeling purposes, continuous VAS-tiredness scores are binarized using the median value of the training set (30 points), yielding a binary fatigue outcome (0 = low fatigue, 1 = high fatigue). The median cut-off is selected to obtain approximately balanced classes and ensure stable model training.

To evaluate the robustness of this definition, sensitivity analyses are performed using two additional thresholds commonly used in prior fatigue research: the upper quartile (top 25%) and the upper decile (top 10%) [27,28,29]. All thresholds are derived solely from the training cohort to avoid information leakage.

In particular, applying the more stringent threshold of VAS-tiredness top 10% results in severe class imbalance, leading to markedly poorer classification performance, especially for the high-fatigue class. Due to this degradation in performance, the median-based binarization is retained as the primary outcome definition. The exact numerical cut-off values and detailed model performance results for all alternative thresholds are summarized in Supplementary Materials Figure S2.

EMA responses were collected between 16:00 and 20:00 each day; responses outside this time window were excluded, and labels were time-stamped at 16:00 for alignment to ensure consistency across samples. Summary statistics of participant-level demographic and clinical characteristics are presented in Table 1.

### 2.2. Data Preprocessing

Wearable-derived and clinical variables are preprocessed to generate participant-level seven-day summary windows. Minute-level heart rate and step data undergo rigorous quality control, with duplicate or physiologically implausible timestamps removed, and are resampled to an hourly resolution. From these cleaned time series, day-wise aggregates are derived to capture short-term autonomic fluctuation (the median of 30-min rolling standard deviations of inter-beat intervals) and overall physical activity levels (total daily step counts).

Clinical and demographic information, including anthropometric indices and work-related variables, are merged by participant ID to represent baseline physiological status and circadian disruption associated with shift work.

Sleep-related variables are derived from wearable device data following a multi-step preprocessing procedure. Raw sleep logs are first quality-checked to remove duplicate timestamps and physiologically implausible durations. Daily main sleep episodes are identified within each 24-h window, and total sleep duration (in minutes) is computed for each participant. From these cleaned daily series, mean sleep duration over the preceding 1-, 3-, and 7-day periods is calculated to represent acute and cumulative sleep debt.

To quantify sleep regularity, each participant’s habitual wake time is estimated as the 7-day rolling average of their wake hour, and deviations from this average are computed across corresponding time windows. Larger deviations indicate greater irregularity in circadian rhythm. All sleep-related variables are merged with clinical and activity features by participant ID after temporal alignment and missing-value imputation. Before model development, samples containing missing values in any of the modeling features or target variables are excluded (listwise deletion). As a result, the final dataset includes only fully observed sensor modalities, and no artificial modality-level missingness is introduced during model development. Of the initial 9877 seven-day observation windows, 334 samples with incomplete data are removed, resulting in 9543 valid windows.

Day-of-week is encoded as a categorical indicator (Monday–Sunday). Current shift status (cur_shift) denotes whether the participant is on shift (1) or off shift (0).

Collectively, these predictors span multiple domains—autonomic activity, physical activity, sleep quantity and regularity, circadian rhythm stability, and demographic/occupational context—and serve as input variables for subsequent modeling analyses. The list and descriptions of derived key features are provided in Supplementary Materials Table S1.

### 2.3. Machine Learning Modeling

All modeling analyses are performed using the preprocessed datasets described above, and participant-level independence is maintained by separating the training and test sets according to individual ID prior to model development. Categorical variables, including sex, occupational group, and shift-work status, are label-encoded, and only numerical features are standardized using z-score normalization with the StandardScaler.

Three supervised learning algorithms are implemented for binary fatigue classification: a PyTorch (v2.1) long short-term memory (LSTM) network, XGBoost (v1.7), and Logistic Regression (LR) implemented using scikit-learn (v1.3). The LSTM model consists of two stacked layers (hidden sizes 64 and 32) with a dropout rate of 0.3, followed by fully connected layers and a sigmoid activation to produce probability outputs. The model is trained with the Adam optimizer (learning rate of 0.001), a batch size of 32, and up to 50 epochs, with early stopping applied based on validation loss (patience set to 10). XGB is trained with 100 estimators, a maximum tree depth of 6, a learning rate of 0.1, and log-loss as the evaluation metric. To introduce stochastic variability and better estimate performance variability across runs, randomization is applied by setting the subsample and colsample_bytree parameters to 0.8. The LR model uses L2 regularization with a maximum of 1000 iterations.

To evaluate model robustness, the entire pipeline is repeated across 30 random seeds (1–30). For each iteration, all models are trained and tested independently, and performance is assessed using accuracy, F1-score, the area under the receiver operating characteristic curve (AUROC), and the area under the precision–recall curve (AUPRC). The mean and standard deviation of these metrics are reported across all 30 runs. We report mean (±SD) across 30 seeds; for SHAP inspection we illustrate one representative run selected by the highest AUROC on the validation split.

### 2.4. Explainability Analysis Methods

Post-hoc analyses are conducted to interpret model predictions and identify the clinical, behavioral (activity and sleep), and demographic relevance of key features in the binary fatigue models. Model interpretability is assessed using SHapley Additive exPlanations (SHAP) for the best-performing LR, XGB, and LSTM models. Global feature importance is quantified by the mean absolute SHAP value per feature, and beeswarm plots are used to illustrate both the ranking and the direction of feature effects on the predicted probability of fatigue.

### 2.5. Selective RAG-Enhanced Hybrid ML–LLM Framework

We develop a hybrid inference framework that integrates an ML model with an LLM to enhance prediction reliability in uncertain cases while maintaining computational efficiency. The LLM is selectively activated only when the ML model’s predicted probability of high fatigue falls within the uncertainty zone (0.45–0.55). To assess the robustness of this threshold selection, we conduct sensitivity analyses using broader uncertainty ranges (0.25–0.75 and 0–1) with Llama 3.3 (70B parameters). When LLM inference is applied to the entire test set (0–1 range), total inference time reaches approximately 910 min. In contrast, restricting LLM activation to the 0.45–0.55 uncertainty range reduces inference time to approximately 162 min, while achieving superior predictive performance. These results indicate that selective LLM activation substantially reduces computational cost while simultaneously improving prediction performance. Predictions with higher or lower confidence (*p* < 0.45 or *p* > 0.55) are directly accepted from the ML model. Detailed results of the sensitivity analyses are provided in Supplementary Materials Table S2.

To support contextual reasoning, a RAG pipeline dynamically retrieved the five most similar training samples for each uncertain test case. All training samples were embedded into a 384-dimensional vector space using a pre-trained sentence transformer (all-MiniLM-L6-v2). Each sample included feature values and metadata (e.g., HRV metrics, sleep duration, step counts, shift status, and day of week), serialized into text format for semantic similarity search based on cosine distance.

Additionally, a rule-based knowledge distillation step is applied to embed explicit symbolic reasoning within the LLM. This process involves using GPT-5 as a teacher model to extract interpretable decision rules from the training data. Specifically, we randomly select one representative participant from each fatigue decile between 10 and 100 to ensure coverage across the full spectrum of fatigue states. For each representative participant, GPT-5 analyzes their feature patterns and generates human-interpretable rules expressed in formal logic notation (>, <, ≥, ≤, and/or). The complete set of extracted rules is provided in Supplementary Materials Figure S3. These rules are incorporated into the LLM prompt under a dedicated block titled “Knowledge Distillation Rules (FOLLOW ABSOLUTELY)” to guide the LLM’s reasoning process and ensure consistency with expert-validated decision patterns.

Each structured prompt contains six components: (1) a task definition, (2) feature descriptions, (3) rule-based knowledge distillation block, (4) RAG-retrieved examples, (5) ML baseline prediction with probability, and (6) test-case features. The LLM returns a JSON-formatted output with three fields—prediction (0 = low fatigue, 1 = high fatigue), confidence (0–1), and reasoning (textual explanation referencing rules or examples).

LLM inference is performed locally to ensure reproducibility and independence from external APIs. We evaluate Llama 3.3 (70B parameters), deployed via Ollama. The inference temperature is fixed at 0.1 to ensure consistent generation behavior, with a 300-s timeout and up to two automatic retries per request. Invalid or partial responses are automatically parsed to extract predictions and confidence values.

To ensure output consistency and reproducibility, the inference temperature was fixed at 0.1 to minimize stochastic variability. The LLM returned structured JSON-formatted outputs containing prediction, confidence, and reasoning fields. The distribution of LLM confidence scores (mean: 0.66 ± 0.10; 85.1% ≥ 0.6) demonstrated stable output reliability across the uncertainty subset. The distribution of LLM confidence scores is presented in Supplementary Materials Figure S4.

**Figure 1:**
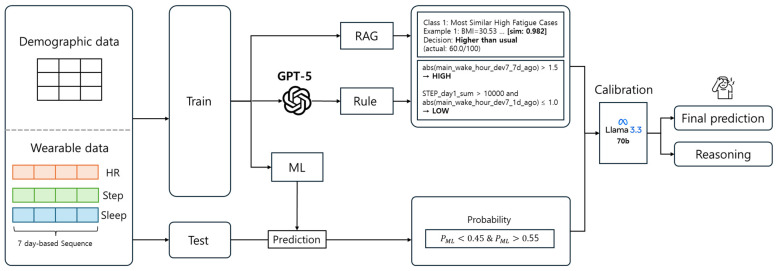
Overview of the Selective RAG-Enhanced Hybrid ML–LLM inference framework. The ML model outputs predictions and selectively triggers the locally hosted LLM only for borderline cases (0.45 ≤ *p*_ML ≤ 0.55). For triggered cases, the LLM prompt is constructed with six ordered components: (1) a task definition outlining the LLM’s role as an expert in wearable-based fatigue assessment, (2) concise feature descriptions providing domain-specific interpretations, (3) a rule-based knowledge-distillation block containing symbolic reasoning rules distilled from GPT-5, (4) RAG-retrieved examples representing the top-k most similar training samples, (5) the ML baseline prediction with probability and binary output to contextualize uncertainty, and (6) test-case features. The LLM returns a JSON-formatted output containing {is_higher_than_usual, confidence, reasoning}. Final predictions are derived through ensemble fusion of ML and LLM outputs using convex combination methods, and all results are logged for calibration and auditability.

After obtaining the LLM decision for the uncertainty subset, we form the final prediction by combining the ML and LLM outputs through several ensemble strategies. We implement and compare multiple post-hoc fusion approaches including confidence-weighted averaging, adaptive override based on confidence thresholds, soft voting, and convex combination methods. In the convex combination approach, the ensemble probability is computed as a weighted average of the ML probability and an LLM-derived pseudo-probability, where the weighting parameter controls the relative influence of each model. The LLM pseudo-probability is constructed by blending the LLM’s categorical prediction (high or low fatigue) with its reported confidence level and the original ML probability. We then compare the ML baseline (restricted to the uncertainty subset) against these ML + LLM hybrid strategies using accuracy, precision, recall, and F1-score. The overall workflow of the selective RAG-enhanced hybrid inference process is summarized in Figure 1.

## 3. Results

### 3.1. Machine Learning Model Performance

Table 2 summarizes the predictive performance of the three machine-learning models—LR, XGB, and LSTM—evaluated across 30 random seed runs on the independent test set. Performance was assessed using accuracy, area under the receiver operating characteristic curve (AUROC), area under the precision–recall curve (AUPRC), and F1-score.

Across models, LR consistently achieved the highest discriminative performance (AUROC = 0.587 ± <0.001; AUPRC = 0.770 ± <0.001), despite its linear simplicity. XGB demonstrated comparable precision–recall characteristics (AUPRC = 0.724 ± 0.007) but slightly lower discrimination (AUROC = 0.518 ± 0.009). The LSTM model, which incorporated temporal dependencies, achieved moderate performance (AUROC = 0.553 ± 0.015) with stable recall (AUPRC = 0.755 ± 0.010), suggesting that sequence modeling captured limited additional variance beyond tabular features.

The performance pattern indicates that the wearable feature space is well-approximated by low-dimensional, near-linear structure, explaining why LR matched or exceeded the deeper/ensemble models. Consequently, classical algorithms such as logistic regression performed as well as—or even better than—deep and ensemble approaches, as the underlying temporal dependencies were already summarized into aggregated day-level statistics

### 3.2. Model Explainability

**Figure 2:**
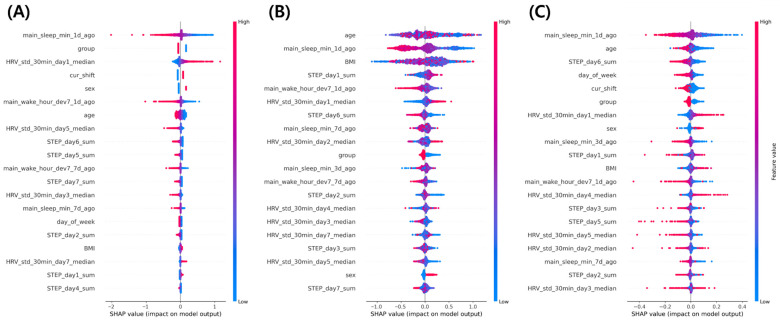
Explainable AI (XAI) analysis of model-specific feature importance. (**A**) Logistic regression summary plot. (**B**) XGBoost summary plot. (**C**) LSTM summary plot Panels (**A**–**C**) show SHAP-based XAI visualizations, where red indicates higher feature values associated with increased predicted fatigue and blue indicates lower values. Features are ranked by mean absolute SHAP value, representing the magnitude of each variable’s contribution to the model output.

SHAP analysis was conducted to interpret feature-level contributions across the three best-performing models—LR, XGB, and LSTM—as summarized in Figure 2. Across all models, sleep-related and autonomic variables consistently emerged as dominant predictors of fatigue, whereas demographic and shift-related features exhibited secondary yet interpretable effects.

For the LR model (Figure 2A), recent sleep duration (main_sleep_min_1 d_ago) emerged as the most influential predictor, with longer sleep associated with lower predicted fatigue probability, reflecting better recovery and rest. In contrast, short-term heart rate variability (HRV_std_30 min_day1_median) showed the opposite pattern, where higher variability (red, right) corresponded to increased predicted fatigue, possibly indicating autonomic instability under acute stress. Current shift status and occupational group also contributed meaningfully, with on-shift workers and police officers exhibiting higher predicted fatigue than off-shift workers and firefighters. Finally, female participants tended to have higher predicted fatigue probability compared with male participants, highlighting potential gender-related differences in fatigue perception.

For the XGB model (Figure 2B), age emerged as the most influential feature, exhibiting a mixed, non-linear relationship with predicted fatigue—both younger and older participants showed variable fatigue probabilities across the distribution. Sleep duration (main_sleep_min_1 d_ago) remained an important factor, with longer sleep associated with lower predicted fatigue, consistent with restorative recovery. BMI also demonstrated a heterogeneous pattern, suggesting diverse physiological effects across individuals. Among behavioral variables, daily step count (STEP_day1_sum) was positively related to predicted fatigue, indicating that higher activity levels contributed to short-term tiredness. In contrast, wake-time deviation (main_wake_hour_dev7_1 d_ago) showed an inverse trend (red, left), with greater circadian irregularity associated with lower predicted fatigue, likely reflecting non-linear model behavior. Finally, short-term heart-rate variability (HRV_std_30 min_day1_median) was positively associated with fatigue, consistent with increased autonomic strain under high-fatigue conditions.

For the LSTM model (Figure 2C), which also presents SHAP-based summary results, the overall ranking of predictors largely paralleled that of the tree-based model, but the importance was more concentrated on features capturing short-term sleep and temporal context—such as day_of_week and cur_shift (current shift status). These recurrent patterns indicate that the LSTM model particularly emphasized short-term fluctuations in sleep duration and work schedule, highlighting the contextual influence of recent sleep–wake and shift patterns on daily fatigue perception.

Overall, these attribution patterns demonstrate that short-term sleep quantity and autonomic balance are the most consistent predictors of fatigue, regardless of model architecture. This convergence across linear, ensemble, and deep learning methods suggests that the wearable-derived feature space primarily encodes structured, quasi-linear physiological patterns rather than complex temporal interactions.

### 3.3. A Selective RAG-Enhanced Hybrid ML-LLM

The final test set included 329 samples located within the uncertain ML prediction region (probability between 0.45 and 0.55), corresponding to approximately 17% of the total testset. This subset displayed a moderately balanced class distribution, with 107 low-fatigue (32.5%) and 222 high-fatigue (67.5%) cases. The baseline ML model (Logistic Regression) achieved an accuracy of 0.556 and an F1-score of 0.659 in this uncertain region, indicating limited discriminative capacity and leaving room for enhancement via selective LLM intervention.

When the LLM was selectively activated for these uncertain samples, it modified the ML prediction in 96 out of 329 cases (29.2%). Among these, 58 changes improved prediction accuracy (ML incorrect → LLM correct), whereas 38 resulted in degradation (ML correct → LLM incorrect), yielding a net gain of +20 correct predictions. The remaining 233 cases (70.8%) were left unchanged, where the LLM agreed with the original ML output, indicating stable convergence between the two models in most uncertain scenarios.

Performance comparison between the ML baseline and the ML + LLM ensembles is summarized in Table 3. Among the four ensemble strategies—Soft Voting, Adaptive, Confidence-Weighted, and Convex Combination—the Convex Combination ensemble achieved the highest performance, with an accuracy of 0.617 and an F1-score of 0.725, corresponding to +6.1 percentage points (pp) in accuracy and +6.6 pp in F1-score relative to the ML baseline. This improvement demonstrates that even limited LLM integration confined to uncertain predictions can meaningfully enhance classification robustness.

The hybrid model primarily improved recall for high-fatigue cases while maintaining stable precision. In the uncertain region (0.45 ≤ *p* ≤ 0.55), performance metrics improved from 0.556/0.684/0.635/0.659 to 0.617/0.703/0.748/0.725 for accuracy, precision, recall, and F1, respectively, and on the full test set from 0.707/0.739/0.918/0.819 to 0.718/0.741/0.927/0.827 (McNemar *p* < 0.05). These results indicate that selective LLM integration can enhance classification robustness in ambiguous regions without sacrificing computational efficiency.

The Convex Combination ensemble outperformed other strategies because it jointly optimized the weighting parameter (α) and classification threshold, allowing adaptation to the class distribution within the uncertainty subset (0.45 ≤ PML ≤ 0.55; 67.5% high-fatigue). In contrast, fixed-threshold approaches (Soft Voting, Adaptive, Weighted) using the default threshold of 0.5 were suboptimal for this skewed subset distribution.

**Figure 3:**
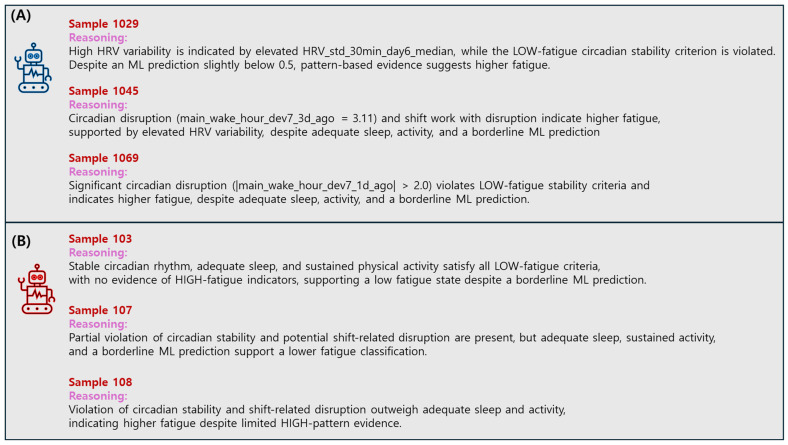
Representative reasoning outputs of the LLM in the selective inference. (**A**) Typical improved case where the LLM corrected an ML misclassification by applying a rule-based trigger and aligning with high-similarity retrieved examples. (**B**) Typical degraded case where the LLM overrode a correct ML prediction due to partial similarity to borderline examples or weak rule activation. Each reasoning output summarizes the predicted label, confidence, and key evidence (activated rules, or retrieved examples) that influenced the decision.

Representative reasoning outputs are illustrated in Figure 3, contrasting improved and degraded cases. In the improved case, the LLM leveraged explicit symbolic rules and retrieved examples to correctly override the ML misclassification. In contrast, the degraded case shows how the LLM occasionally over-generalized from borderline examples when rule conditions were weakly satisfied. These examples demonstrate how the rule-augmented reasoning process enhances transparency by making explicit the evidence paths leading to each prediction.

## 4. Discussion

This study demonstrates the feasibility and effectiveness of a Selective RAG-Enhanced Hybrid ML–LLM framework for wearable-based fatigue prediction in real-world occupational settings. By integrating a fast and interpretable ML backbone with selective LLM reasoning, the proposed approach overcomes key challenges of uncertainty and interpretability that commonly arise in sensor-based mental health monitoring. Specifically, the system activates the LLM only for samples in the ML uncertainty zone (0.45 ≤ *p* ≤ 0.55), thereby improving both prediction robustness and computational efficiency. Compared with the baseline ML model, the Convex Combination ensemble achieved superior performance, particularly in recall for high-fatigue cases—a clinically valuable trait in fatigue surveillance for emergency responders.

The rule-based reasoning patterns, derived independently through symbolic rule extraction, showed substantial alignment with the dominant features identified by SHAP-based post-hoc interpretation of the ML models, despite the absence of any SHAP-derived cues in the LLM prompts. Both approaches highlighted irregular wake-time deviations, short sleep duration, and shift-work interactions as key drivers of high fatigue, aligning with the interpretation of circadian instability and sleep deprivation as major contributors. Similarly, HRV-related rules exhibited the same directionality as SHAP, with higher variability corresponding to higher predicted fatigue, reflecting autonomic imbalance under high-fatigue conditions.

In contrast, a partial discrepancy was observed for step-related rules, where inactivity (low steps) was associated with fatigue in the symbolic rules, whereas SHAP attributed higher fatigue probability to higher activity levels. This divergence likely reflects nonlinear model behavior in the activity domain. Notably, such inconsistencies were partly compensated by RAG-retrieved examples, which provided additional contextual grounding for the LLM’s reasoning in ambiguous cases.

Overall, the strong alignment among symbolic rules, RAG-contextual reasoning, and SHAP-based associations reinforces that all three modalities converge on the same physiological axes—sleep continuity, circadian regularity, and autonomic balance—as the core determinants of daily fatigue, consistent with prior research identifying these factors as fundamental [30,31].

Across linear (LR), ensemble (XGB), and temporal (LSTM) architectures, convergent SHAP patterns highlighted the dominance of sleep and circadian features, suggesting that these factors encode the most stable low-dimensional representations of fatigue states. The selective LLM integration further refined this predictive landscape by leveraging symbolic reasoning rules and dynamically retrieved examples to interpret ambiguous patterns that ML models alone could not resolve.

From a methodological perspective, the rule-augmented RAG design illustrates a practical strategy for achieving explainable reasoning without model fine-tuning. By distilling symbolic rules derived from domain-informed GPT-5 outputs into the LLM prompt, the hybrid framework merged explicit logic-based inference with contextual example retrieval. This not only improved interpretability—by making the reasoning process transparent through activated rules and retrieved analogues—but also stabilized LLM behavior across queries, addressing a common limitation of stochastic generative responses. The resulting hybrid reasoning process provided traceable decision pathways that can be audited or clinically validated, which is critical for translational deployment in health-monitoring contexts.

A notable strength of this framework lies in its explainable AI (XAI) capability. Even when the LLM’s prediction diverged from the ML baseline, the reasoning trace—explicitly expressed through activated rules and retrieved examples—allowed human interpreters to understand why an incorrect or unexpected decision occurred. This transparency transforms model errors from opaque outcomes into interpretable evidence trails, enabling domain experts to audit, contextualize, and iteratively improve model logic. By combining interpretability, adaptability, and computational efficiency, the proposed framework demonstrates a balanced paradigm for trustworthy AI in human-centered health applications.

To preliminarily assess the clinical utility of the explanations, we conducted a small-scale expert verification by sampling representative cases from the uncertainty region. One board-certified psychiatrist reviewed the rule activations and RAG-retrieved examples for these cases. The clinician confirmed that the explanations were clinically meaningful and helpful for understanding borderline predictions, suggesting that the framework’s interpretability may support real-world clinical decision-making. However, a comprehensive user study with multiple domain experts will be warranted to systematically evaluate explanation quality and clinical applicability.

Despite these promising results, several limitations should be noted. The present study focused on a relatively homogeneous occupational group of emergency responders, which may limit generalizability to broader populations or other fatigue-related contexts. Although the selective LLM integration improved interpretability and robustness, the framework still depends on the underlying ML model’s feature space; unseen sensor modalities or extreme data missingness could challenge its reasoning reliability. Furthermore, the symbolic rules distilled from limited samples may capture only partial behavioral patterns, underscoring the need for iterative refinement with larger and more diverse datasets.

A further limitation is that we did not evaluate robustness under modality-level missingness—such as the loss of HRV or sleep features—because the analysis relied on complete-case preprocessing. Future work will incorporate explicit missing-data simulations to better reflect real-world wearable conditions.

## Figures and Tables

**Table 1:** Participant-level demographic and clinical characteristics.

Characteristics	Train Set (n = 237)	Test Set (n = 60)	Total (n = 297)	*p*-Value
Age (years)	43.73 (9.22)	41.67 (7.95)	43.31 (9.00)	0.112
BMI * (kg/m^2^)	25.17 (3.37)	25.01 (3.25)	25.14 (3.34)	0.688
Height (cm)	171.38 (7.78)	170.24 (6.80)	171.15 (7.59)	0.352
Weight (kg)	74.27 (12.89)	72.79 (12.03)	73.97 (12.72)	0.382
Sex				0.436
Female	52 (21.9)	16 (26.7)	68 (22.9)	
Male	185 (78.1)	44 (73.3)	229 (77.1)	
Cur shift *				0.126
Yes	113 (47.7)	22 (36.7)	135 (45.5)	
No	124 (52.3)	38 (63.3)	162 (54.5)	
Occupation				0.285
Police	85 (35.9)	26 (43.3)	111 (37.4)	
Firefighter	152 (64.1)	34 (56.7)	186 (62.6)	

Values are expressed as mean ± standard deviation for continuous variables and as number (percentage) for categorical variables. *p*-values were calculated using the Mann–Whitney U test for continuous variables and the chi-square test for categorical variables. No significant differences were observed between the training and test sets, indicating balanced participant characteristics across data splits. * Abbreviations: BMI, body mass index; Cur shift, current shift work status.

**Table 2:** Performance of machine-learning models across 30 random seed runs.

Model	Accuracy	AUROC *	AUPRC *	F1-Score
Logistic Regression	0.707 (<0.001)	0.587 (<0.001)	0.770 (<0.001)	0.819 (<0.001)
XGBoost	0.653 (0.008)	0.518 (0.009)	0.724 (0.007)	0.774 (0.006)
LSTM *	0.640 (0.023)	0.553 (0.015)	0.755 (0.010)	0.758 (0.025)

Values are reported as the mean ± SD of model performance across 30 independent random seed runs, evaluated on the independent test set. * Abbreviations: AUROC, Area Under the Receiver Operating Characteristic curve; AUPRC, Area Under the Precision-Recall Curve; LSTM, Long Short-Term Memory.

**Table 3:** Performance comparison between the baseline ML model and the Selective RAG-Enhanced Hybrid ML-LLM framework.

Test Set	Method	ACC *	PRE *	REC *	F1 *
Full test set (n = 1863)	ML (LR *)	0.707	0.739	0.918	0.819
	ML + LLM (Soft *)	0.709	0.741	0.915	0.819
	ML + LLM (Adaptive *)	0.677	0.742	0.848	0.791
	ML + LLM (Weighted *)	0.685	0.743	0.862	0.798
	ML + LLM (Convex *)	0.718	0.741	0.937	0.827
Uncertainty subset only (n = 329)	ML (LR)	0.556	0.684	0.635	0.659
	ML + LLM (Soft)	0.562	0.699	0.617	0.656
	ML + LLM (Adaptive)	0.386	0.635	0.211	0.371
	ML + LLM (Weighted)	0.431	0.680	0.297	0.414
	ML + LLM (Convex)	0.617	0.703	0.748	0.725

Performance metrics (accuracy, precision, recall, and F1) were evaluated both for the entire test set and for the ML-uncertainty subset (0.45 ≤ P_ML_ ≤ 0.55). The hybrid ensemble integrated LLM judgments only for samples within the uncertainty region, showing performance improvement over the ML baseline particularly in the convex combination ensemble configuration. * Abbreviations: Acc, accuracy; PRE, precision; REC, recall; F1, F1-score; LR, logistic regression; Soft, soft ensemble; Adaptive, adaptive ensemble; Weighted, confidence-weighted ensemble; Convex, convex combination ensemble.

## Data Availability

The data presented in this study are not publicly available due to privacy and ethical restrictions.

## Supplementary Materials

The following supporting information can be downloaded at: https://www.mdpi.com/article/10.3390/bioengineering13010058/s1, Supplementary Materials Figure S1: Cohort selection flow and exclusion criteria; Supplementary Materials Figure S2: Sensitivity Analysis of Alternative Fatigue Classification Thresholds; Supplementary Materials Table S1: Overview of key feature definitions and construction methods; Supplementary Materials Table S2: Sensitivity Analysis of Uncertainty Thresholds for Selective LLM Activation; Supplementary Materials Figure S3: Symbolic rule sets distilled from the GPT-5 teacher model; Supplementary Materials Figure S4: Distribution and reliability of LLM confidence scores in the uncertainty region.

## Abbreviations

The following abbreviations are used in this manuscript:

AUROC	Area Under the Receiver Operating Characteristic Curve
AUPRC	Area Under the Precision–Recall Curve
ACC	Accuracy
F1	F1-score
BMI	Body Mass Index
PP	Percentage Points
PRE	Precision
REC	Recall
SD	Standard Deviation
LR	Logistic Regression
XGB	Extreme Gradient Boosting
LSTM	Long Short-Term Memory
ML	Machine Learning
LLM	Large Language Model
RAG	Retrieval-Augmented Generation
SHAP	SHapley Additive exPlanations
EMA	Ecological Momentary Assessment
HRV	Heart Rate Variability
