# Supplementary material for: A Selective RAG-Enhanced Hybrid ML-LLM Framework for Efficient and Explainable Fatigue Prediction Using Wearable Sensor Data
Source: Bioengineering (Basel). 2026 Jan 3;13(1):58. doi: 10.3390/bioengineering13010058 (PMC12838294; doi:10.3390/bioengineering13010058)
Supplement: Supplementary file 1 [file bioengineering-13-00058-s001.zip › bioengineering-4001729-supplementary.pdf]

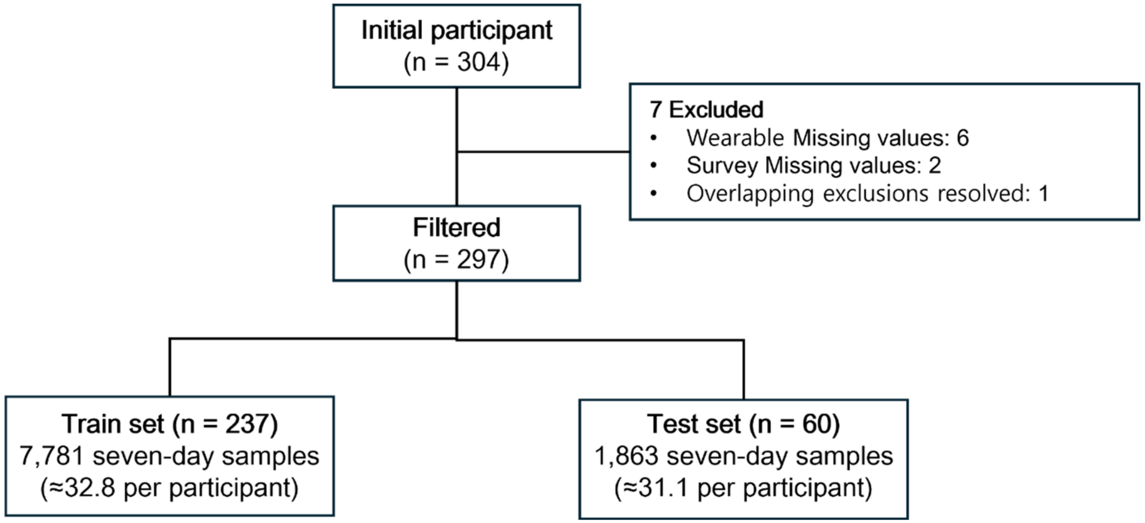

Supplementary Materials Figure S1. Cohort selection flow and exclusion criteria

Of the initial 304 participants, seven were excluded due to missing data, resulting in 297 participants included in the analysis. Data were split into training (n = 237, 7,781 samples) and test (n = 60, 1,863 samples) sets for model development and validation.

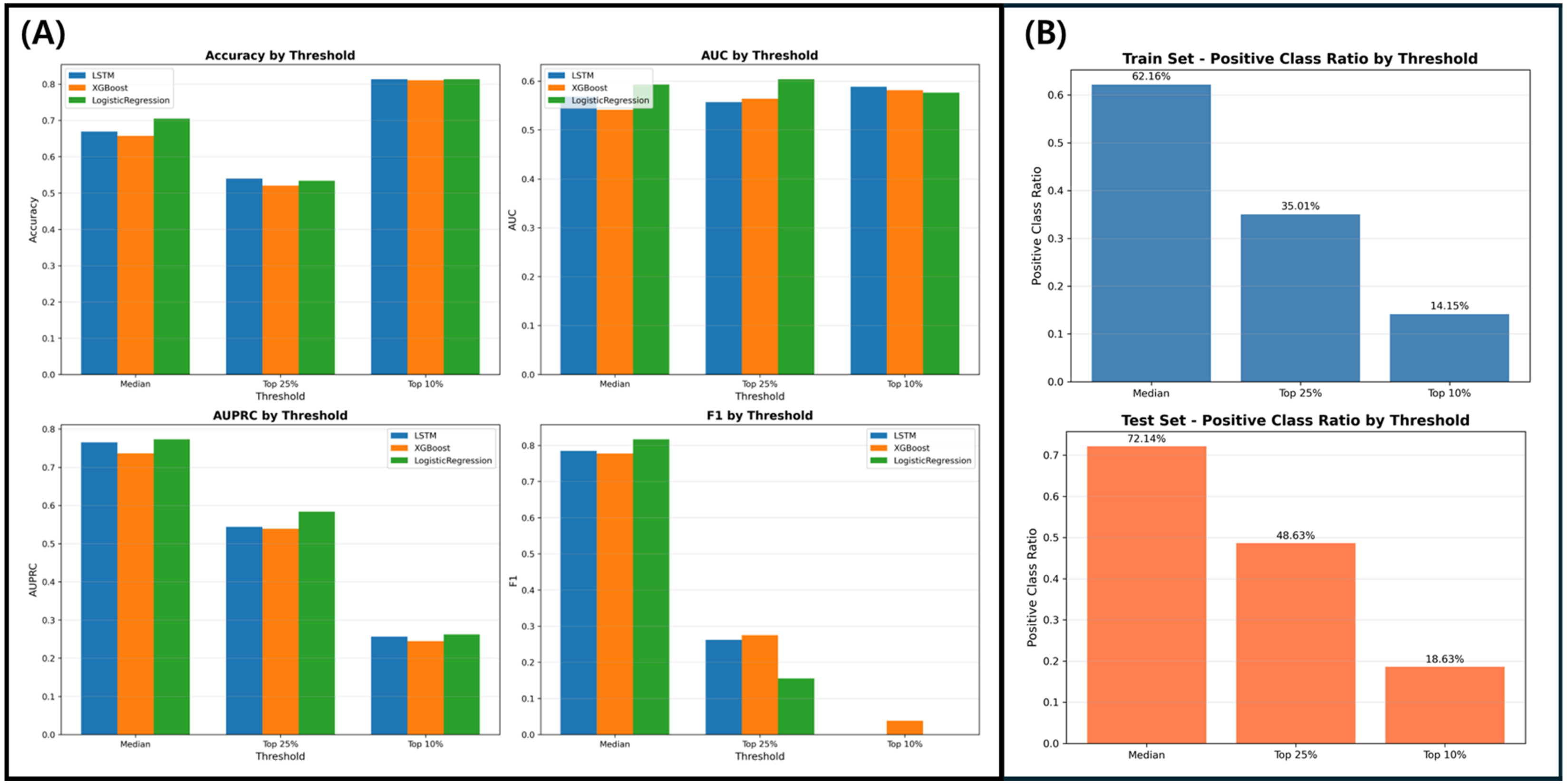

Supplementary Materials Figure S2. Sensitivity Analysis of Alternative Fatigue Classification Thresholds

To evaluate the robustness of the primary fatigue (outcome) definition, we conducted a comprehensive sensitivity analysis using three binarization criteria based on the VAS-tiredness distribution in the training set: (1) median (50th percentile), (2) top 25% (75th percentile), and (3) top 10% (90th percentile). All thresholds were computed exclusively from the training cohort to prevent information leakage. Model performance evaluation was conducted across Logistic Regression (LR), XGBoost, and LSTM architectures using 30 independent random seeds.

Supplementary Materials Table S1. Overview of Key Feature Definitions and Construction Methods

Category	Feature Name	Description	Computation Method
HRV	HRV_std_30min_day1–7_median	Heart Rate Variability standard deviation in 30-min windows — reflects autonomic nervous system balance over the previous 7 days	30-min rolling SD of interbeat intervals, median per day (days 1–7 before prediction)
Sleep	main_sleep_min_1d/3d/7d_ago	Main sleep duration (minutes) – indicates recent sleep quantity and quality	Sum of sleep epochs per night, averaged over 1-, 3-, and 7-day windows
	main_wake_hour_dev7_1d/3d/7d_ago	Deviation of wake-up time from 7-day habitual mean – represents circadian rhythm stability	Wake time difference (day i – 7-day rolling mean)
Activity	STEP_day1–7_sum	Total step count – represents daily physical activity level	Daily total steps aggregated per day for 7 days before prediction
Temporal	day_of_week	Day of week (0 = Monday, 6 = Sunday) — captures workload and recovery cycle variations	Extracted from timestamp
	cur_shift	Current work shift (0 = non-shift, 1 = shift)	Derived from self-report

Features with suffixes such as “dayX” or “Xd_ago” indicate the temporal distance from the prediction day (e.g., *day1* = 1 day ago). Sleep-related variables (main_sleep_min and main_wake_hour_dev7) were clustered into 1-, 3-, and 7-day representative patterns to capture temporal regularity.Baseline variables (BMI, age, sex, group, cur_shift) remain static across all windows.

Supplementary Materials Table S2. Sensitivity Analysis of Uncertainty Thresholds for Selective LLM Activation

Test set	Method	ACC*	PRE*	REC*	F1*	Inference Time
Full range (n=1863)	ML (LR*)	**0.707**	**0.739**	**0.918**	**0.819**	910 min 32.8 sec
	ML+LLM (Soft*)	0.684	0.740	0.865	0.798	
	ML+LLM (Adaptive*)	0.646	0.750	0.764	0.757	
	ML+LLM (Weighted*)	0.531	0.744	0.534	0.622	
	ML+LLM (Convex*)	0.604	0.764	0.651	0.703	
Uncertainty range [0.25 – 0.75] (n=1714)	ML (LR)	**0.702**	0.735	**0.918**	**0.819**	841 min 52.9 sec
	ML+LLM (Soft)	0.674	0.736	0.851	0.789	
	ML+LLM (Adaptive)	0.641	0.749	0.751	0.750	
	ML+LLM (Weighted)	0.501	0.734	0.495	0.591	
	ML+LLM (Convex)	0.590	**0.762**	0.621	0.684	
Uncertainty range [0.45 – 0. 55] (n=329)	ML (LR)	0.556	0.684	0.635	0.659	**161 min 31.9 sec**
	ML+LLM (Soft)	0.562	0.699	0.617	0.656	
	ML+LLM (Adaptive)	0.386	0.635	0.211	0.371	
	ML+LLM (Weighted)	0.431	0.680	0.297	0.414	
	ML+LLM (Convex)	**0.617**	**0.703**	**0.748**	**0.725**	

This supplementary material presents a sensitivity analysis evaluating different uncertainty ranges for selective large language model (LLM) activation within the hybrid ML–LLM inference framework. We compare three uncertainty thresholds—0.45–0.55, 0.25–0.75, and 0–1—using Llama 3.3 (70B parameters) to assess the trade-off between prediction performance and computational cost. The results demonstrate that restricting LLM activation to the 0.45–0.55 uncertainty window yields the most favorable balance between performance gain and inference efficiency, while broader ranges lead to diminishing returns with substantially increased computational overhead. Predictions outside the uncertainty region are directly accepted from the ML model.

For vas_tiredness_bin = 1 (high fatigue)
Rule 1. (abs(main_wake_hour_dev7_7d_ago) > 1.5) = 1
Rule 2. (abs(main_wake_hour_dev7_1d_ago) > 1.5 or abs(main_wake_hour_dev7_3d_ago) > 1.5) = 1
Rule 3. (cur_shift = 1 and abs(main_wake_hour_dev7_3d_ago) > 1.0) = 1
Rule 4. (main_sleep_min_1d_ago < 300 and main_sleep_min_7d_ago < 300) = 1
Rule 5. (STEP_day5_sum = 0 or STEP_day6_sum = 0 or STEP_day7_sum = 0) = 1
Rule 6. (HRV_std_30min_day6_median > 7.5 and HRV_std_30min_day1_median > 4.5) = 1
Rule 7. (age ≥ 40 and cur_shift = 1 and abs(main_wake_hour_dev7_7d_ago) > 1.0) = 1

For vas_tiredness_bin = 0 (low fatigue)
Rule 8. (STEP_day1_sum > 10000 and STEP_day2_sum > 9000
and abs(main_wake_hour_dev7_1d_ago) ≤ 1.0 and abs(main_wake_hour_dev7_3d_ago) ≤ 1.0) = 0
Rule 9. (main_sleep_min_1d_ago between 380 and 540 and main_sleep_min_3d_ago between 350 and 500
and abs(main_wake_hour_dev7_1d_ago) ≤ 1.0) = 0
Rule 10. (cur_shift = 0 and group = 0 and STEP_day1_sum ≥ 15000) = 0
Rule 11. (STEP_day1_sum < 4000 and main_sleep_min_1d_ago ≥ 200
and abs(main_wake_hour_dev7_1d_ago) ≤ 1.0 and cur_shift = 1) = 0
(seen once; weak signal)

Notes (so you can tweak thresholds fast)
- "abs(...dev...)" means absolute deviation from habitual wake time; large deviations (e.g., > 1–1.5 hours) tended to pair with high fatigue (=1).
- Extremely low recent sleep on multiple windows (e.g., 1d & 7d both < 300 min) also aligned with =1.
- Late-week zeros in steps (days 5–7) showed up with =1.
- High steps (≥ ~10k) with stable wake time deviations (≤ ~1h) pointed to =0.
- These are heuristics from the small sample you pasted—use them as starting rules; you can promote/demote any line after a quick check on the full dataset.

Supplementary Materials Figure S3. Symbolic rule sets distilled from the GPT-5 teacher model

Each rule represents interpretable behavioral or physiological conditions separating low- and high-fatigue states relative to the median cutoff (VAS = 30). The rule sets were used to construct the Knowledge Distillation Rules (FOLLOW ABSOLUTELY) block in the LLM prompt.

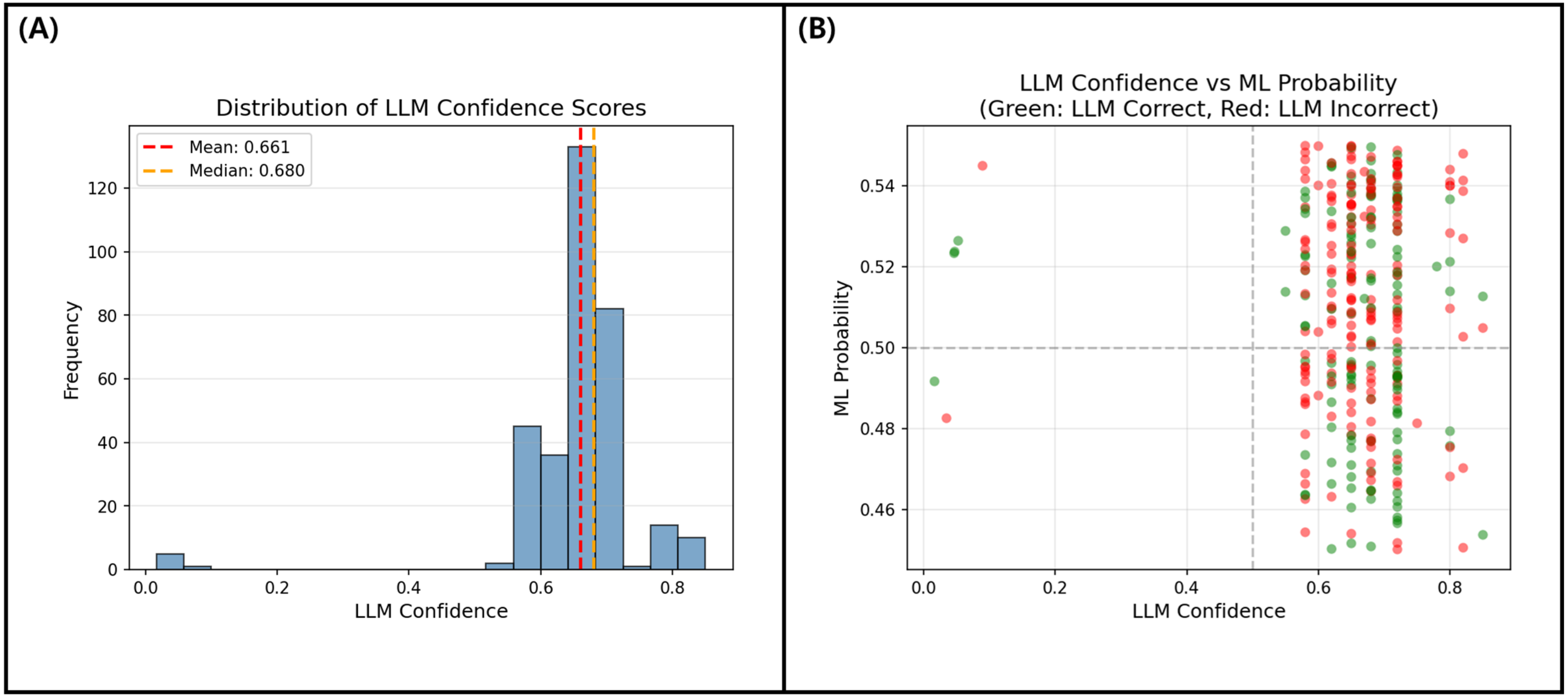

Supplementary Materials Figure S4. LLM output consistency analysis in the hybrid ML–LLM framework

To assess the consistency and reliability of locally hosted LLM (Llama 3.3, 70B parameters) outputs, we analyzed the distribution of confidence scores returned by the LLM for samples within the ML uncertainty region ($0.45 \leq P\_ML \leq 0.55$). (A) Histogram of LLM confidence scores ($n = 329$). The mean confidence was 0.66 ± 0.10 (range: 0.02–0.85), with 85.1% of predictions demonstrating high confidence (≥ 0.6), indicating stable LLM judgments despite the inherent uncertainty of the input samples. Red and orange dashed lines indicate the mean and median, respectively. (B) Relationship between LLM confidence and ML predicted probability. Each point represents a single sample, colored by LLM prediction accuracy (green: correct; red: incorrect). The inference temperature was fixed at 0.1 to minimize stochastic variability.
